# Is a Hospital Pageant Practicable?

**Published:** 1921-05-14

**Authors:** Philip A. Inman

**Affiliations:** Secretary of the Hospital Saturday Fund.


### THE EDITOR'S LETTER-BOX.

### IS A HOSPITAL PAGEANT PRACTICABLE?

*To the Editor of* The Hospital.

Sir,—The suggestions contained in the leader of last week's issue of The Hospital referring to the inauguration of a Hospital Pageant are worthy of the careful attention of all who are interested in the future welfare of the Metropolitan hospitals. Something must be done to destroy the apathy and indifference of the large percentage of people who, at the moment, neither know nor care how, why, or where the hospitals exist. If the voluntary principle is to be preserved, "collective publicity and propaganda constantly carried out are necessary." It would be folly to allow matters to drift until the State assumes control. Now is the time for action.

A Hospital Carnival and Pageant would be valuable from at least two standpoints—it would attract the attention of, and be the means of extracting contributions from, the public. There is a curious ignorance prevalent to-day concerning the work of our hospitals, and any step that will enlighten and educate is both desirable and necessary. Nothing appeals to the popular mind like pageantry. The City of Dublin discovered that in 1913, when a famous pageant was held; the whole history of nursing was shown, beginning with Dorcas and passing all through the years with Saint Helena, Abbess Hilda, Queen Philippa, Florence Nightingale—not forgetting Sairey Gamp and Betsy Pring—types of what might have been. What Dublin did in 1913 London could more than equal in 1921, with all the history of the European War to work upon.

Why confine the effort to one day? Personally I would like to see a "Hospital Week" established, backed by the three central funds and supported by the individual hospitals, "The Week" to include the recognised collecting days, "Hospital Sunday," "Hospital Saturday," and "Our Day." Let the week commence on "Hospital Saturday" with the suggested Carnival and Pageant. This would prepare the way for "Hospital Sunday," when collections would be taken in all places of worship. During the week following public meetings and concerts might be arranged, and addresses delivered on the work and needs of the hospitals. I venture to think that interest and enthusiasm would be created which might solve the present problem of money-raising and be the means of placing the hospitals upon a sound financial foundation.

A note of warning is necessary. In our zeal let us be careful not to arrange dates or details until the question has been thoroughly discussed. A pageant requires much thought and preparation, and it would be most regrettable if the first London hospital venture of this kind were to fail for want of time to make the adequate arrangements. On the other hand, unnecessary delay would be equally regrettable.

Is it not possible to elect a small representative committee to consider the question from all standpoints?—Yours faithfully,

Philip A. Inman,
Secretary of the Hospital Saturday Fund.

*To the Editor of* The Hospital.

